# Germline genome modification through novel political, ethical, and social lenses

**DOI:** 10.1371/journal.pgen.1009741

**Published:** 2021-09-09

**Authors:** Vicki Xafis, G. Owen Schaefer, Markus K. Labude, Yujia Zhu, Soren Holm, Roger Sik-Yin Foo, Poh San Lai, Ruth Chadwick

**Affiliations:** 1 Centre for Biomedical Ethics, Yong Loo Lin School of Medicine, National University of Singapore, Singapore, Singapore; 2 Centre for Social Ethics and Policy, Department of Law, School of Social Sciences, University of Manchester, Manchester, United Kingdom; 3 Center for Medical Ethics, HELSAM, Faculty of Medicine, University of Oslo, Oslo, Norway; 4 Cardiovascular Research Institute, National University Health Systems, Centre for Translational Medicine, Singapore, Singapore; 5 Genome Institute of Singapore, Singapore, Singapore; 6 Department of Paediatrics, Yong Loo Lin School of Medicine, National University of Singapore, Singapore, Singapore; 7 School of Social Sciences, Cardiff University, Cardiff, United Kingdom; Stanford University, UNITED STATES

## Abstract

Much has been written about gene modifying technologies (GMTs), with a particularly strong focus on human germline genome editing (HGGE) sparked by its unprecedented clinical research application in 2018, shocking the scientific community. This paper applies political, ethical, and social lenses to aspects of HGGE to uncover previously underexplored considerations that are important to reflect on in global discussions. By exploring 4 areas—(1) just distribution of HGGE benefits through a realist lens; (2) HGGE through a national interest lens; (3) “broad societal consensus” through a structural injustice lens; and (4) HGGE through a scientific trustworthiness lens—a broader perspective is offered, which ultimately aims to enrich further debates and inform well-considered solutions for developments in this field. The application of these lenses also brings to light the fact that all discussions about scientific developments involve a conscious or unconscious application of a lens that shapes the direction of our thinking.

## Introduction

Gene modifying technologies (GMTs) refer to the array of technologies available to edit an organism’s genome. GMTs allow for the addition, removal, substitution, or modification of a genetic material that affects gene expression and include techniques using base editors and nucleases such as transcription activator–like effector nucleases (TALENs), zinc-finger nucleases (ZFNs), as well as clustered regularly interspaced short palindromic repeats/CRISPR associated protein (CRISPR/Cas)-based systems [1]. There are many applications for these technologies such as for diagnostics and genetic screening, generating animal disease models, drug screening, organ transplantation, etc. Here, we focus on therapeutic applications where the benefits in correcting disease or pathogenic phenotype have been demonstrated from both preclinical and clinical studies using in vitro and in vivo gene therapy approaches on somatic cells [2–4]. Although there is also ongoing research on gene editing of germline cells, it was not known to be carried out on viable human embryos until 2018 when He Jiankui revealed to the world the first clinical research application of human germline genome editing (HGGE) [5]. The significant global controversy created by He’s experiment has stimulated fervent discussions and has prompted us to reflect on the wider context of GMTs especially with respect to HGGE.

This paper examines a range of diverse issues around HGGE through a number of underexplored lenses relating to political, ethical, and social considerations. Viewing an issue through a particular lens allows us to identify dimensions of an issue that may previously not have received adequate attention or that may have been completely obscured due to a focus on different aspects of the issue. For example, the topic of “broad societal consensus” has been written about at length since 2015 [6–10] in an attempt to gain greater support for the concept from all involved in scientific, legal, ethical, and societal contributions to the field, which, in turn, influences policymakers. This concept has been deployed as a central condition for applications of HGGE to proceed. It is therefore important that the concept be firmly based in a realistic context rather than seemingly aspiring to an outcome that has no realistic hope of eventuating. The concept has not been scrutinized from a structural injustice perspective, which illuminates the considerable impediments to ever achieving “broad societal consensus.”

In the manner described above, this paper illuminates new dimensions, brings to light a richer array of pertinent facets, and enables reflection on the matters explored from a different perspective or vantage point. A lens can be broad and relate to a whole discipline or school of thought (e.g., a political, ethical, and realist lens) or much narrower to focus on a specific area or social phenomenon (e.g., examining an issue through a scientific trustworthiness lens) within a larger context. While we are cognizant that somatic editing for gene therapy also raises social justice and nationalism issues, we have chosen a narrower lens to focus on HGGE as the latter deserves closer scrutiny due to its ability to make heritable changes in the human genome.

While lenses can frame the topic in diverse ways, this consideration of the issue in our work is not intended to function in ways that framing manipulations do when used in the media (for example, to shape the readership’s views and attitudes by concealing some aspects of the issue) or in research (for example, to elicit a set of responses in a particular context) [11]. Rather, the explicit articulation of the lenses through which the issue is considered in this work invites the reader to consider these issues from a new, perhaps unfamiliar, perspective. In this way, discussions acquire greater depth and can broaden in useful ways, which, cumulatively, might lead to approaches that remove certain impediments and facilitate solutions.

The HGGE issues examined in this work are situated within political, ethical, and social domains to which narrower lenses are applied to illuminate facets previously underexplored. The range of diverse issues considered through different lenses in this paper includes the just distribution of HGGE benefits through a realist lens; HGGE through a national interest lens; “broad societal consensus” in HGGE through a structural injustice lens; and HGGE through a scientific trustworthiness lens.

A related paper in this issue, Kaan and colleagues, considers regulation and oversight over HGGE interventions with potential for heritable changes in the germline. The Kaan and colleagues paper also aims to promote reflection, specifically with regard to current and proposed approaches to regulation and oversight considerations.

### Realism about the application of HGGE

#### Social justice

Numerous documents urge us to remain at least vigilant about and monitor potential social divisions, injustices, and exacerbating inequities resulting from the implementation from HGGE [12–15]. Some take an even stricter approach requiring that HGGE should proceed/be permitted only if its application does not exacerbate existing social divisions [12,13]. For instance, the Nuffield Council’s 2018 report on genome editing and human reproduction articulated this requirement by stating that HGGE “should be permitted only in circumstances in which it cannot reasonably be expected to produce or exacerbate social division or the unmitigated marginalisation or disadvantage of groups within society” [16,17]; an official opinion of France’s Comité Consultatif National d’Ethique on the Ethical Challenges of Gene Editing states that “an ethical requirement to avoid exacerbating inequalities in human social development” [18]; and a discussion paper by New Zealand’s Royal Society Te Apārangi states that we need to “prevent uses [of gene editing] which reinforce prejudice and worsen inequalities within and between societies” [19]. However, serious questions remain about both of these approaches. The first approach, which requires us merely to monitor developments, leaves open what is to be done if increasing social divisions, injustice, or exacerbating social divisions are observed. Monitoring the emergence of HGGE-caused inequality and social division is good practice—but the call for monitoring itself provides no guidance on what to do when problematic developments occur. The second approach, requiring certain constraints, equally provides no guidance about how such developments should be addressed if they take place. Indeed, it should be noted that this second approach cannot be interpreted overly stringently: Some inequalities resulting from the use of HGGE would have to be tolerated. To appreciate this, consider an HGGE intervention offered free of charge to prospective parents to enable them to have genetically related, healthy children. Many parents would take up this intervention—but some may not. As a result, there will be some inequality between children coming about as a result of the intervention and those who do not. Similarly, there will be inequality between parents whose offspring is afflicted by the genetic condition and those who are not. As this example shows, it would be implausible to require that no inequalities may result from the application of HGGE. This suggests that a more moderate interpretation of the proposal should be implemented, where inequalities are permitted but not those that lead to social division or increased disadvantage of entire groups.

Answers on how to deal with such scenarios are required and must be included in reports that seek to comprehensively provide the conditions under which HGGE may, if at all, proceed. Some might counter that such social justice concerns are exaggerated: As with any novel medical technology, the cost of gene modification will decrease over time, making access to them widely available. It is, however, conceivable that gene modification could “lock in” a plethora of advantages that other factors, for example, education, cannot. Lee Silver has, for instance, predicted a future divide between the gene rich and the gene poor [20]. Others have also imagined fragmentation of human beings into different species [21]. However, these extreme scenarios might be misleading as they envisage the widespread adoption of HGGE by socially advantaged groups. What is needed is guidance for more realistic scenarios in which some degree of increased inequality to access to HGGE is likely.

What can such guidance look like? One possible approach would be to leverage on the difference between a potential and an actual Pareto-optimal social change, emerging from making HGGE available. A social change is Pareto optimal if everyone affected by the change is at least as well-off after the change as before. A truly Pareto-optimal change may increase inequality, but does not harm anyone. A potential Pareto-optimal change is a change where some gain and some lose, but those who gain, gain enough to be able to compensate those who lose for their loss. Social changes that are not at least potentially Pareto optimal are often objectionable. An actual Pareto-optimal change is a scenario in which those who gain actually do compensate those who lose, with the result that, in the end, no individual is worse off, and at least some individuals are better off than before the change took place. There may, in the future when it comes to be possible, be many uses of HGGE that are potentially Pareto optimal, for instance, modifications that increase intelligence and thereby advantage the individual in relation to, e.g., university admission. These are probably most likely to be used by and benefit those who are already socioeconomically advantaged. However, unless compensation actually takes place, a potentially Pareto-optimal change will deepen social inequalities. The guidance to policymakers would therefore ensure that those who benefit from gene modification adequately compensate those who stand to lose and would consider in advance what to do if they cannot be compensated for.

#### Collective action problem

Genome modification of humans and other species can also create collective action problems; when individual actors act in a way that is rational for them, it will have overall negative effects for all. In the human context, collective action problems may occur when one country allows a particular form of gene modification that is prohibited elsewhere. The possibility of reproductive or therapeutic travel exports the effects of its liberal regulation to more restrictive ones. This is not necessarily innocuous, as illustrated by the current use of sex selection methods in assisted reproduction [22,23]. A country in which parents mostly do not have strong sex preferences for their children may be able to allow sex selection without any harmful effects on the sex ratio, but it will, at the same time, make it possible for affluent parents from societies that do not allow sex selection because of strong societal sex preferences to gain access to and use the techniques. In relation to HGGE, collective action problems can occur if a particular modification creates protection against an environmental risk factor and thereby reduces the incentive to remove the risk factor itself. These kinds of collective action problems are difficult to handle, as is demonstrated by the largest current collective action problem at the present time, i.e., climate change, because their resolution requires coordinated action at the international level and because they require some sacrifice of national interest.

### National interest lens

Ethical debates on GMTs typically consider and weigh up the impact of GMTs on individuals regardless of their nationality. In contrast, those who fund the research and development of GMTs see promotion of national interests (over and above the interests of the individual, but limited to denizens of a given nation) as a key justification for pursuing research and investment into GMTs. Bioethicists have tended to ignore national interest considerations. However, recent developments in gene editing highlight the need to critically engage with the idea of national interest and nationalism, which may play an important role in motivating such research.

National interest is a guiding principle in science policy around the world. Under the Clinton administration in the 1990s, the United States explicitly advocated for “Science in the National Interest” [24], an approach that reverberates today. In a similar vein, by way of example, the Australian Government has established a national interest test for public research funding [25], while Singapore focuses its research funding on “strategic areas of national relevance, to address national needs, prepare for future challenges, and generate new opportunities for growth” [26] (p. 8).

In the context of GMTs, it has been suggested that China has been pursuing research in this area in part to further national interest [5]. A prescient analysis by Jiang and Rosemann [27] observes that gene editing research in China is driven by 3 factors—economic opportunity, medical progress, and novel technological advancement, all as means to broader national ends. For example, economic opportunity is a further means to economic development, social stability, and wide public benefit. Medical progress is seen as having salutary effects on population health, which, for gene editing, includes preventing transmission of heritable diseases. Technological advancement aligns with a national shift toward an innovation society, and China appears to have explicitly identified GMTs as an avenue for promoting global competitiveness, precipitating a “gene editing rush” [27]. Meanwhile, others argue that the US should fund GMT research to compete with China and other countries because this would be in the national interest of the US [28]. GMT research may then be part of broader innovation race between the US and China [29].

When Jiang and Rosemann’s analysis was first published in March 2018, it was limited to basic embryonic research. Yet, only months later, He Jiankui revealed he had produced the world’s first live birth of gene-edited babies in China. The reaction from Chinese authorities was strong and condemnatory, but some who know He, such as William Hurlbut, have argued that he was operating within the implicit expectation for scientists to push boundaries and promote national competitiveness [30]. Nonetheless, in December 2019, He was found guilty of engaging in “illegal medical practice,” which related to him being “a person who has not obtained a medical license but engages medical activities without authorization,” in this case the clinical trial [31].

Promoting the well-being of a nation’s people is one of the central justifications for the state’s existence in the first place [32]. However, national interest intersects with the more contentious notion of nationalism [32], here understood as the idea that it is permissible or even obligatory for countries to prioritize, often aggressively, their own national interest over the interests of other countries or people. For example, high-income countries have used advance purchase agreements to guarantee priority vaccine access to their own citizens, thus depriving poorer nations’ access [33]. The aggressive pursuit of vaccines to promote the health of one’s own nation with little regard for other nations has been termed “vaccine nationalism” and has been condemned as unethical, self-defeating, and reckless [34].

In the context of GMTs, then, the question becomes whether a country may pursue its own national interests in relation to GMT research, at the expense of the interests of others. Viewing GMTs through the lens of national interests helps shed light on the actual reasons GMTs are pursued in some contexts. At the same time, because it plays such a substantial role in political decision-making, it is worth interrogating whether nationalism is a sound reason to pursue research and development of GMTs. This question in turn has 2 components, one normative and the other empirical.

The normative component revolves around a deep question of political philosophy: whether nationalism is justifiable in this context. Here, it is useful to distinguish between nationalism, which is “voluntarist,” thus allowing members to choose their nationality or “organic” where membership is determined by “birth into a collective” and more likely to result in ethnocentric, authoritarian systems developing [35]. Relatedly, some have dismissed “techno-nationalism,” the “hoarding and exploitation of technological advantage by nation states” (p. 113), as inherently problematic in the GMT context and an obstacle to good international governance [17].

Nations arguably do have special obligations toward their own citizens because state funding for GMT research and development is tax funded, and governments have coercive powers of regulation and oversight only over those within their own borders. On standard representative democratic theory, the state must answer directly to its own citizenry [36] (rather than the global population) to justify its coercive powers over them.

The pursuit of national interest must have limits, however. An extreme form of nationalism that completely discounts foreign or global interests is ethically untenable. Even if nations may legitimately prioritize the interests of their citizens, the interests of those living abroad are still ethically relevant and must, at some level, be taken into consideration [37,38]. This is especially so when activities within one nation negatively affect those outside it.

Lax GMT regulation in order to promote innovation may be just such a case. A policy survey of 106 countries found that while no country explicitly permits HGGE, some countries have exceptions to prohibitions or indeterminate regulations that potentially could be interpreted as permitting HGGE under certain circumstances; other countries lacked easily ascertainable regulations, which could be due to a lack of restrictions on HGGE in certain jurisdictions [39]. Laxer regulation in some countries could attract individuals from other jurisdictions with stricter regimes to obtain services there. Those individuals would then return to their home countries, which would potentially bear the costs of GMT failures (for example, through health systems needed to treat deleterious effects). This sort of venue shopping is not merely hypothetical: The first reproductive use of mitochondrial replacement therapy occurred in Mexico largely because of the lead scientist’s perception of a comparatively lax regulation there [40,41]. More broadly, in the information age, research conducted in one country will easily cross borders, and technologies developed will be made available for use and misuse far and wide.

The empirical component of whether GMTs are in a country’s national interest will depend on a host of complex factors and will shift over time. There is a purely economic question of whether GMTs will become profitable enterprises, since many novel interventions fail to live up to their initial hype. But even monetary benefits must be weighed up against other costs, not the least of which is reputational. In He’s case, while the very earliest media coverage in China framed the births as a historic breakthrough [5], subsequent local and international outcry quickly led to regulatory reforms in China to more tightly oversee and restrict HGGE [42].

Even if one is suspicious of nationalistic motivations in the modern political climate, it is undeniable that these play a role in actual national decision-making [29]. While countries will be guided by other considerations as well (including local values and norms), the explicit place national interests hold in the science policy surrounding GMTs requires careful attention. The intersection of these normative and empirical challenges suggests the need for substantially more engagement with the notion of public interest in active discussions and debates surrounding GMTs, including at a global level beyond national borders.

### “Broad societal consensus” through a structural injustice lens

“Broad societal consensus” in the context of HGGE relates to the inclusion of “perspectives from multiple nations” and “a wide range of perspectives and expertise” as well as “consensus about what is (or is not) at stake, what risks do (or do not) warrant immediate concern, and what common ground is needed to achieve shared and mutually acceptable endpoints for scientific and technological intervention.” [43]. The concept broad societal consensus is to be distinguished from “informed adaptive consensus“ proposed in Kaan and colleagues, a related paper in this issue, with the latter referring specifically to a scientific and ethical consensus among experts. To date, particularly, “broad societal consensus” and other forms of public engagement/discussions have not been viewed through a structural injustice lens, an important consideration, since structural inequalities exclude from discussions and public engagement large segments of the global population. For many years, calls for broad societal consensus have been prominent, but reference to this requirement should finally permanently be abandoned because when issues of structural injustice are considered, it becomes evident that “broad societal consensus” can be neither fully inclusive nor representative of the global community. Use of this term risks making those most unable to be involved invisible.

The call for “broad societal consensus” was first made in 2015 [6]. Despite subsequent persistent efforts over many years to clarify and justify the concept [7–9,43], some have more recently ruled out global consensus due to its practical infeasibility [44]. There is, however, agreement that “broad public dialogue” should be fostered and promoted internationally and by individual countries [45] and that public engagement should be promoted within the systems and infrastructure available [46]. In fact, most recent publications call for some form of public engagement in discussions and debates around gene editing, and, more specifically, HGGE [47–49]. Repeatedly, however, public engagement practices in affluent countries such as the US, the United Kingdom, Denmark, and France have been held up as models [46], thus overlooking the realities in most parts of the world.

A “global observatory for gene editing” was suggested as a mechanism to serve several functions among which would be a platform for convening meetings and international discussions that would consider power imbalances and issues of inclusion [50]. This proposal adopts a multipronged approach to address the complexities of rapid global activity and advances in the field, but it remains unclear how it could achieve truly inclusive and broad global participation. In addition, a recent statement replaces “broad societal consensus” with discussions of “public empowerment” stressing the need for organized civil society to have a voice at the global table [51].

On the surface, such proposals appear to be globally, socially, culturally, and religiously inclusive models of science communication that are empowering and equitable [52]. In substance, however, they are not and cannot be so unless the people excluded and the reasons for their exclusion are, at the very least, acknowledged. Typically excluded are groups impacted by (local and global) structural inequities, which arise from biases and preferential standing for some, while certain groups are systematically excluded; this preferential standing enables (and the exclusion correspondingly disables) the development of capabilities, which make possible one’s involvement and equal participation in society, and prevents exploitation and marginalization [53,54]. Such exclusionary structures are deeply embedded in all aspects of civil society, including policies, practices, and even the behaviors of those privileged by such structures, who consciously or unconsciously support their perpetuation [53,54].

The circumstances in which people are born and live their lives interact in complex ways, and, ultimately, influence and shape people’s ability, or not, to engage in spheres of human activity such as education, employment, healthcare, as well as broader pursuits such as science. An example of the cascading effects of structural inequality is witnessed through the impact of a lack of education on health and well-being throughout life [55] and on people’s ability to secure employment [56]. People’s employment status, in turn, impacts on their health and well-being in significant ways, as well as their ability to contribute to other areas of life. Similar issues impact some immigrants in high-income countries, who often work long hours in low-paid jobs; this leaves very little time for engagement in broader scientific pursuits [52]. As may be expected, people with “unformed” views and those least engaged in science are individuals with the lowest household incomes and lowest educational attainment, and their personal circumstances impact on their ability to ascertain the relevance of science in their lives [57].

We are advised in relation to HGGE that “… we **all** (author emphasis) have a shared responsibility to think carefully about our future and how we might want to direct it.” [58]. However, the majority of the global population does not enjoy the privileges of those who have an education, a job, a secure and safe home to live in, food on the table at every meal, and absence of major violence or instability in their lives arising from a number of threats, such as warfare, persecution, famine, entrenched poverty, homelessness, disease, exploitation, or extreme global events such as pandemics. For example, millions of people are displaced due to conflicts [59], and, according to an International Committee of the Red Cross statement, 2 billion people were impacted by “fragility, conflict or violence” in late 2018 [60]. The number of undernourished people has been increasing over the years and stood at around 821 million people in 2018, and there is evidence that climate change is already impacting on food security [61], thus painting a very grim picture for the future. Prior to the pandemic, 689 million people were classified as living in extreme poverty (i.e., 9.2% of the global population in 2017 living on less than $1.90 a day), while 24.1% of the global population lived on less than $3.20 a day and 43.6% on less than $5.50 a day in the same year, all of whom are also considered poor. A 2020 estimate calculated that an additional 88 to 115 million people would fall into extreme poverty, as a result of the pandemic, climate change, and conflict, thus sadly raising the total living in extreme poverty to an approximate figure of 703 to 729 million people [62]. The day-to-day struggle to sustain oneself and one’s family, experienced by billions around the world, automatically precludes involvement in decisions about HGGE and the future of humanity given how precariously their own future in the world is placed [63].

A realistic and nuanced consideration of “broad societal consensus” and calls for broad and inclusive deliberation on HGGE that do not give the impression of full inclusivity is necessary. The European Group on Ethics in Science and New Technologies Ethics of Genome Editing has also adopted the term “broad societal consensus” but further clarified the categories of individuals it considers necessary for inclusion: “… with special attention to representatives of women’s rights, rights of the child, gender equality, social equality, reproductive rights and justice, disability rights, and human rights in general” [64] (p. 36). Even though the term “broad societal consensus” remains highly problematic, consideration of representatives of such groups is encouraging. A recent proposal for broad inclusion of citizens of many nations comes in the form of a global citizens’ assembly to deliberate on genome editing [65]. One of the aims of such a global assembly being developed is “to generate a global conversation about genome editing” [66]. This is a laudable and urgently needed “discussion,” which does not exaggerate the purpose to claim that global consensus will be achieved.

Taking into account local and global structural inequalities, which often impede any form of involvement, is a first small step toward helping to reduce these, where possible. Silence on such matters cannot be the foundation on which we advocate for broad societal consensus or any other form of broad inclusive discussions/public engagement in relation to human HGGE and science more broadly. To remain silent on who cannot be included is to obscure their existence and to inadvertently promote the value of those of us fortunate enough to have a voice in such important decisions.

### Scientific trustworthiness lens

Numerous codes of research ethics articulate the values and commitment that researchers are expected to abide by and promote [67–70] (for a more complete list of national research ethics guidelines and codes, see https://council.science/what-we-do/freedoms-and-responsibilities-of-scientists/ethical-responsible-conduct). Scientists have a responsibility to maintain the trustworthiness of their profession and bring unethical experiments to light, when they learn of them. Many institutions where scientists carry out their research have set up whistleblowing policies, so that concerns about misconduct, irregularities, or malpractices can be taken seriously and investigated fully. However, maintaining trustworthiness goes beyond general obligations all of us may have to report wrongdoing. Scientists rely on public trust to secure funding, attract participants, assuage fears about innovations, and, ultimately, facilitate implementation of important discoveries. This is not a mere exercise in public relations to get the public to blindly accept whatever scientists say or do. Rather, scientists hold a special privileged position in society as they guide the world in scientific advancements that protect and improve lives by enhancing the human experience and serve the broader needs of society. They are entrusted with public funds to achieve their work, but in recognition of their role in society and the impact of their work, they have specific social responsibilities. Scientists must therefore earn and maintain the public’s trust by acting responsibly within the profession, including taking steps to identify wrongdoing and put a stop to it when it occurs, particularly in response to fast-paced technological developments.

In terms of policy, the 2010 Singapore Statement on Research Integrity issued at the Second World Conference on Research Integrity states that “researchers should report to the appropriate authorities any suspected misconduct” [71]. Similarly, the 2013 Montreal Statement on Research Integrity in Cross-Boundary Research Collaborations, developed as part of the Third World Conference on Research Integrity, advises that “collaborating partners should promptly take appropriate action when misconduct or other irresponsible research practice by any partner is suspected or confirmed” [72]. Both documents provide general guidance but lack detail on the process.

In international research where the researcher who becomes aware of potential grave breaches is a collaborator, there is a clearer reporting pathway, as detailed in a rare dedicated guidance document [73] in relation to breaches of the Australian Code for the Responsible Conduct of Research [74]. However, reporting pathways are lacking for international research where the researchers who learn of unethical experiments are not collaborators and may themselves reside outside the jurisdiction where the experiment is occurring, may be unfamiliar with those countries’ standards, and may lack confidence in the appropriate action to be taken.

Some combination of these factors was evidently in play in the He Jiankui case. While the experiment prompted considerable global outcry when it was announced, a number of scientists and other colleagues around the world were informed in advance about the experiment, but did not publicly raise the issue [75]. These individuals were undoubtedly placed in a difficult position by virtue of the information disclosed to them, which they may have preferred never to have received. In this sense, they were implicated in the scandal without invitation. However, once information was received, even if unsolicited, these individuals as bystanders had an unenviable ethical dilemma to resolve: remain silent (and suffer the consequences) or reveal details of the experiment (and suffer the consequences)? They chose the former. Given how swiftly He’s experiment was shut down after news reports about it first broke, earlier revelations might have prevented the unethical experiment from proceeding as far as it did.

There is, therefore, a need for reflection among those working on GMTs concerning current approaches to preventing unethical, unprofessional conduct. Existing, institutional, and country-level mechanisms may be inadequate, thus requiring a *trans*-national approach [76]. Any such efforts, however, must take into account the pragmatic difficulties of international coordination efforts. Further reflection on relational obligations that scientists have as a result of the position they hold in society is also always valuable.

## Conclusions

We have brought into focus political, ethical, and social issues around HGGE, which have often been absent from discussions and which, when examined more closely, may illuminate the landscape and assist in developing more informed outlooks, arguments, and, ultimately, decisions. The application of the 4 lenses (realist, national interest, structural injustice, and scientific trustworthiness) leads not only to a number of conclusions but also to considerations about the use of the lenses themselves.

In 2 of the 4 cases (i.e., Realism about the application of HGGE and “Broad societal consensus” through a structural injustice lens), looking through the lenses revealed inadequacies of current discourse surrounding GMTs: First, requirements that inequalities not be increased are unrealistic, and, second, calls for broad societal consensus neglect structural injustice. Indeed, in relation to the latter, we suggest that a call for such a requirement be permanently abandoned. As regards the former, our conclusion is less demanding but still strong: Realism is needed about what is possible in the light of actual social (dis)advantages and collective action problems.

Use of the national interest lens and the scientific trustworthiness lens leads to more subtle conclusions: In the first case, the need for deeper reflection on the role of national interest in science policy, its relationship to nationalism, and where the moral limits to pursuing national interest should be set; in the second, the desirability of reflection about the special ethical responsibilities of scientists, such as drawing attention to issues of concern about unethical conduct, while having eyes open to realistic possibilities internationally.

What, if anything, have we learned about the use of the lenses in general? Lenses can be not only unenhanced but also “rose-tinted” (i.e., not realist) and also both wide angle and zoom. A wide-angle lens broadens our gaze, arguably appropriate when considering national interest; a zoom lens can expose in detail the implications of specific issues such as social inequities. In the absence of an objective “view from nowhere,” it is clear that we are always looking through some lens and that it is thus equally, if not more, important to be aware of our own often unrecognized use of lenses as it is to critically analyze the perspectives of others. What we have shown here are the ways in which the explicit adoption of a lens can be revealing of viewpoints that are frequently taken for granted in debate or completely overlooked to the detriment of achieving optimal solutions.
